# Detection of the Frail Elderly at Risk of Postoperative Sepsis

**DOI:** 10.3390/ijerph20010359

**Published:** 2022-12-26

**Authors:** Antonio Sarría-Santamera, Dinara Yessimova, Dmitriy Viderman, Mar Polo-deSantos, Natalya Glushkova, Yuliya Semenova

**Affiliations:** 1 Department of Medicine, Nazarbayev University School of Medicine, 010000 Astana, Kazakhstan; 2 Agency for Health Technology Assessment, Institute of Health Carlos, 28029 Madrid, Spain; 3 Department of Epidemiology, Biostatistics and Evidence Based Medicine, Al-Farabi Kazakh National University, 050040 Almaty, Kazakhstan

**Keywords:** sepsis, frail elderly, hospital frailty risk score, elixhauser comorbidity index, Spain

## Abstract

With the increase in the elderly population, surgery in aged patients is seeing an exponential increase. In this population, sepsis is a major concern for perioperative care, especially in older and frail patients. We aim to investigate the incidence of sepsis in elderly patients receiving diverse types of surgical procedures and explore the predictive capacity of the Hospital Frailty Risk Score (HFRS) to identify patients at high risk of incidence of postoperative sepsis. This study relies on information from the Spanish Minimum Basic Data Set, including data from nearly 300 hospitals in Spain. We extracted records of 254,836 patients aged 76 years and older who underwent a series of surgical interventions within three consecutive years (2016–2018). The HFRS and Elixhauser comorbidity index were computed to determine the independent effect on the incidence of sepsis. Overall, the incidence of postoperative sepsis was 2645 (1.04%). The higher risk of sepsis was in major stomach, esophageal, and duodenal (7.62%), followed by major intestinal procedures (5.65%). Frail patients are at high risk of sepsis. HFRS demonstrated a high predictive capacity to identify patients with a risk of postoperative sepsis and can be a valid instrument for risk stratification and vigilant perioperative monitoring for the early identification of patients at high risk of sepsis.

## 1. Introduction

Aging is an increasing public health issue as extended longevity has resulted in an aging population in most developed countries. Eurostat forecasted that by 2080, in the European Union, the proportion of the population aged 65 years and older will reach 30% [1]. In Spain, aging progresses at an unstoppable rate: there are 125 people over 64 for every 100 under 16, around 17% of the total population, and approximately 25% of older people are octogenarians. It was projected that by 2050 the number of octogenarians will constitute more than four million people or more than 30% of the total elderly population [2].

Extended longevity brings an increase in age-associated health problems [3]. This new epidemiological landscape led to the growth in the need for surgical interventions in very old people, who on many occasions are frail [4,5]. In recent years, surgery in the elderly has undergone steady improvement due to advances in different surgical and anesthetic techniques, but above all due to a better understanding of aging [6]. All of this made it possible to expand the surgical offerings to patients of even very advanced ages [7,8].

Frailty is a condition that may be related to aging, resulting in a reduction in vital reserves, resistance to stressors, and a high risk of adverse outcomes of surgical procedures [9]. Frailty is present in about 10% of people aged 65 and increases up to 50% in people aged over 85 [10,11]. Frailty is associated with increased risk of sepsis, which is a life-threatening organ dysfunction provoked by an impaired response to infection. Sepsis is a significant concern in perioperative care, more specifically in elderly and frail patients. Surgical interventions are common in older patients but are associated with a significant risk of complications and other adverse clinical outcomes, such as sepsis [12]. Sepsis is associated with increased mortality, prolonged hospital stays, increased intensive care requirements, need for additional surgeries, and increased costs: sepsis mortality exceeds 30% and, in the elderly, it could be as high as 50% [13].

The data on the rates of postoperative sepsis in elderly population are needed to tailor the preventive strategies. Additionally, it is important to determine whether frailty, a measure of physiologic reserve and vulnerability in older patients [14], is predictive of adverse clinical outcomes after surgery. In this national study, we aim to investigate the incidence of sepsis in elderly patients receiving diverse types of surgical procedures in Spain and to explore the predictive capacity of the Hospital Frailty Risk Score (HFRS) [15] to identify the risk of incidence of post-surgical sepsis.

## 2. Materials and Methods

### 2.1. Study Design and Procedures

This study relies on information from the Spanish National Hospital Discharge Database (SNHDD), which includes data on patients discharged from all Spanish hospitals admitting patients covered by the publicly financed National Health System in Spain [16]. The SNHDD is an administrative database managed by the Spanish Ministry of Health that collects information from all private and public hospitals. These hospitals are required by law to provide data from all subjects hospitalized for at least 24 h. The following variables for each patient are included in the SNHDD: age, sex, place of residence, dates of admission and discharge, discharge destination, primary diagnosis, secondary diagnosis (up to a maximum of 19), and procedures (therapeutic or diagnostic) conducted during the hospitalization period (up to a maximum of 20). To codify diagnoses and procedures, the SNHDD applies the International Classification of Disease Tenth Revision (ICD-10). Details on the SNHDD can be found online [17].

From this dataset, we extracted all records of patients aged 76 years and older who underwent a series of surgical interventions within three consecutive years (from 1 January 2016 to 31 December 2018). The following diagnosis-related groups (DRG) were analyzed: 220 major stomach, esophagus, and duodenal procedures, 221 intestine procedures; 228 hernia procedures, 263 haparoscopic cholecystectomies, 301 hip replacements, 302 knee replacements, and 308 traumatic hip procedures. From the MBDS we retrieved the following information: sex, age, year of hospital admission and length of hospital stay, admission to intensive care unit (ICU) and its length, and concomitant diseases.

The primary outcome variable was the incidence of postoperative sepsis, which was defined based on Patient Safety Indicators developed by the Agency for Healthcare Research and Quality (AHRQ) [18] using specific ICD-10 codes reported as complications for each hospitalization. Information on survival outcomes was grouped as survived or died.

### 2.2. Evaluation of Sepsis Risk Indices

Two risk indices were evaluated: Hospital Frailty Risk Score (HFRS), and Elixhauser Comorbidity Index (ECI) [19]. HFRS has been proposed and outlined by Gilbert and co-workers as an alternative to the existing frailty scores. This score was especially developed for older individuals in acute care settings as they tend to have diagnoses associated with frailty and are characterized by high resource use. According to the authors, all patients are categorized as having low frailty risk (score < 5), intermediate frailty risk (score 5–15), or high frailty risk (score > 15). HFRS relies on ICD-10 codes and can be calculated based on routinely collected data. HFRS identifies the risk of frailty and healthcare-related outcomes, e.g., death and/or hospital readmissions [20]. The HFRS was previously developed and validated in a British cohort of older people. This score was obtained based on comorbidities reported at admission for each patient included in the database (see Table S1 for itemized scoring criteria based on ICD-10 codes).

ECI is a score based on ICD-10 scores that can be identified from abstracted hospital data. ECI is used to categorize comorbidities present during episodes of hospitalization and medical services provided. Like HFRS, ECI can be used to forecast the need for hospital resources and predict in-hospital mortality [21].

### 2.3. Statistical Analysis

Prior to statistical analyses, we evaluated the type of data distribution with the help of the Kolmogorov–Smirnov test. Since the distribution of data were non-normal, we presented the quantitative variables as medians with the interquartile range (IQR). Qualitative data were presented as absolute numbers and their percentages and statistical significance were assessed by the Pearson’s χ^2^ test. Multivariate regression analysis with computing unadjusted and age- and sex-adjusted odds ratios (OR) with 95% confidence intervals (CI) was performed to evaluate the incidence of sepsis and identify the independent effects of HFRS and Elixhauser. Area Under the Curve (AUC) ROC curves were obtained to assess the performance of the instruments’ predictive capacity to explain the incidence of sepsis in multivariable logistic models.

## 3. Results

The general characteristics of the study population are summarized in Table 1, where 254,836 patient cases were analyzed, of which 2645 patients had sepsis reported as a complication.

Table 2 presents the incidence of postoperative sepsis in selected DRGs. Knee replacement was characterized by the lowest incidence of sepsis (0.09%), followed by hernia repair and hip replacement (0.24 and 0.35%, respectively). In contrast, the patients underwent major stomach, esophageal, and duodenal procedures as well as major intestinal procedures had the highest incidence of postoperative sepsis (7.62 and 5.65%, respectively).

Table 3 shows the main characteristics of patients receiving the different interventions analyzed in this study. The highest median HFRS was seen in the patients who underwent trauma hip procedures (4.9 (IQR 3.5–7.6)) and hip replacement (4.3 (IQR 1.4–6.4)). Likewise, Table 3 indicates how the higher risk of sepsis was in major stomach, esophageal, and duodenal procedures (7.62%), followed by major intestinal procedures (5.65%). Patients with hip fractures were the oldest (median age of 86 years). The median age was 84 years for hip replacement and ranged between 79 and 81 years for the other procedures. As for the ECI, the highest median scores were seen in patients with major stomach, esophageal, and duodenal procedures, and major intestinal procedures (3 (IQR 2–4)). Nearly half (48.28 %) of all septic patients had major intestinal procedures, followed by laparoscopic cholecystectomy (15.95 %). 

Table 4 shows the adjusted and unadjusted OR of the specific effect of HFRS to estimate the incidence of sepsis for each of the different surgical procedures analyzed.

Table 5 and Table 6 reflect the results of multivariable logistic regression analysis and AUC comparing the predictive capacity as estimated by HFRS measure either as a continuous variable or in specific categories. For this, we applied two approaches to the categorization of HFRS. According to approach A, HFRS was classified as 0.1–4.9, 5.0–9.9, and ≥10. Meanwhile, according to approach B, HFRS was categorized as 0.1–1.7, 1.8–4.9, and ≥5.0. Both approaches to categorization of HFRS resulted in similar predictive effects given the similar AUC values (0.937 vs. 0.938).

**Figure 1:**
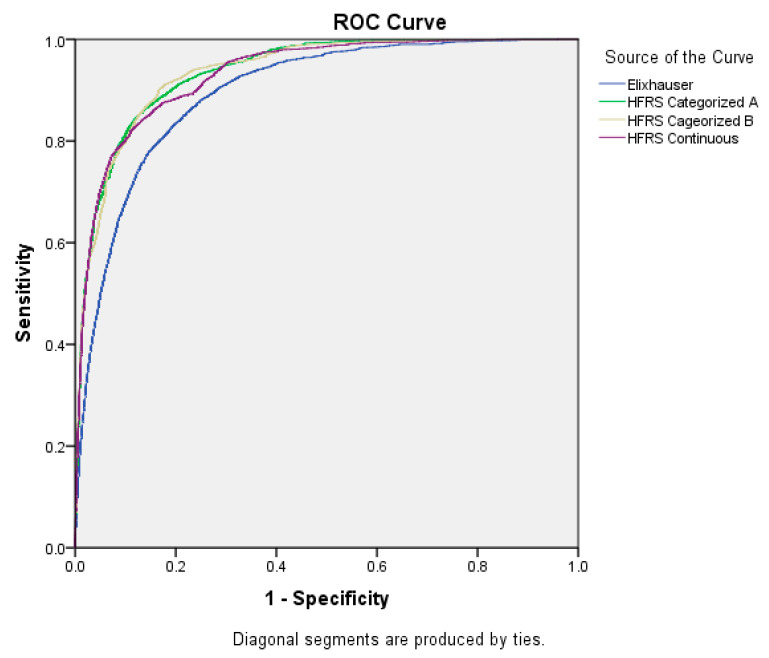
Area Under the Curve for the different parameters considered: separate multivariable models for Elixhauser Comorbidity Index and HFRS.

**Figure 2:**
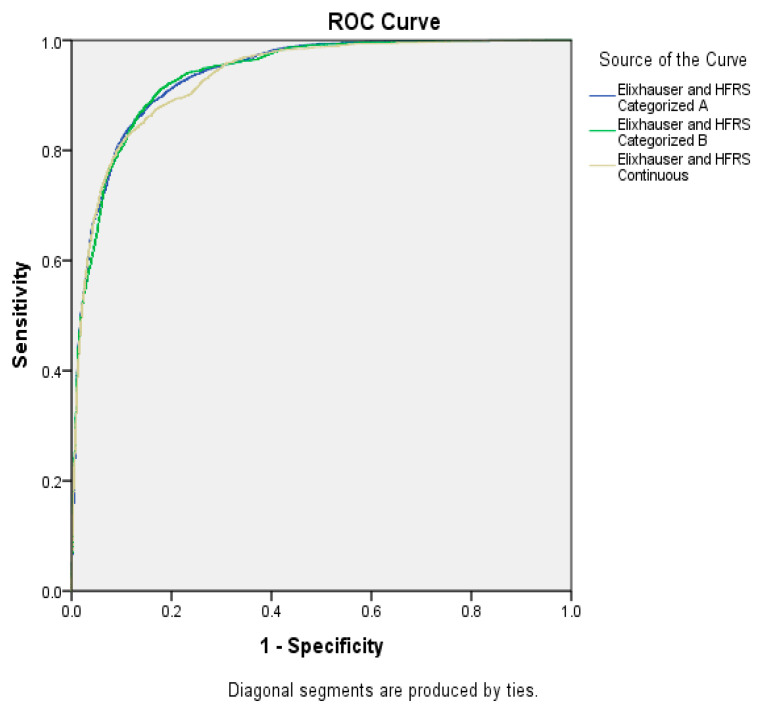
Area Under the Curve for the different parameters considered: including Elixhauser Comorbidity Index with HFRS to predict postoperative sepsis.

AUC did not improve with the inclusion of EIC in any of the different models assessed (Figure 1 and Figure 2).

## 4. Discussion

This national study was aimed at the investigation of the incidence of sepsis in elderly patients undergoing different surgical procedures in Spain and at exploration of the predictive capacity of HFRS to identify the risk of incidence of post-surgical sepsis. HFRS appears to be a valid scoring system for risk stratification of frail elderly patients requiring surgical interventions and may be used for their vigilant perioperative monitoring and initiation of timely treatment. Our results suggest that higher comorbidity on admission, measured through higher EIC and frailty, measured through HFRS, were associated with a significantly higher incidence of post-surgical sepsis in elderly patients. However, HFRS showed better discrimination than EIC, as reflected by the respective AUC. Incidence of postoperative sepsis was significantly associated with intra-hospital mortality and a state of critical illness. Major abdominal procedures, especially intestinal procedures, were more likely to lead to sepsis.

Postoperative sepsis accounts for one-third of all sepsis cases. Although recent decades bear witness to advances in the management of sepsis, resulting in decreasing mortality, the morbidity associated with sepsis imposes a substantial burden on healthcare services. According to a recent study performed in the USA, 30-day mortality constituted 9.6%, while 33% of patients developed chronic critical illness: at 12 months, septic patients had worse outcomes, with persistent severely impaired performance status and increased mortality (41.4%). Another study from the USA reported a trend of increase in the rates of severe surgical sepsis, which persisted even after adjustment for all relevant covariates. Older sepsis survivors are also suffering from even higher disability and long-term mortality after hospital discharge [22].

An earlier study on the incidence of severe sepsis in the population of Madrid reported an annual rate of 14.1/10,000 inhabitants, the highest for individuals aged 84 and older (230.8/10,000) [23]. Another study on the epidemiology of sepsis in Spain showed that incidence increased with age. The case-fatality rate was also higher in older patients as compared with their younger counterparts [24]. Both studies reported on high costs associated with the incidence of sepsis. Like polytrauma, acute myocardial infarction, or stroke, early identification, and appropriate management in the initial hours after sepsis develops improved outcomes [25].

Surgical patients are vulnerable to sepsis and other infectious complications during hospitalization. Advanced age critical increases the risk of surgical sepsis [26,27,28]. Several possible explanations could be proposed to explain this observation, including emergency situations, comorbidities, polypharmacy, steroid administration, poor nutritional status, or immune disturbances. These factors may contribute to more complicated perioperative care, a higher frequency of adverse outcomes, which might convert into increased postoperative mortality [29,30,31,32]. All those problems exhibit a higher frequency in the elderly and much higher in the case of those suffering from frailty syndrome.

Management of surgery-associated infections is always challenging, but elderly frail patients have more complex demands. When surgical intervention is needed, interdisciplinary intervention, anesthesiology, internal medicine, geriatrics, nutrition, etc. are required [33] focusing on early identification of surgical sepsis and proper management to reduce suffering and saving lives [34].

This study has several limitations: the major one comes from its retrospective design. Additionally, due to unavailability of data we were not able to measure outcomes other than mortality as the long-term health consequences of sepsis survivors can be severe. Moreover, there was a lack of data regarding the type of surgical intervention-emergency vs. scheduled surgical procedures. Still, this study has several strengths and the most important one is a large sample size with inclusion of all patients aged 76 years and older who underwent a surgical intervention during the study period. Moreover, to the best of our knowledge, this study is the first to explore the predictive capacity of HFRS as a risk factor for the incidence of postoperative sepsis in senile patients. More research may be necessary to confirm these initial findings as well as to identify the possibility to select a HFRS cut-off points for risk stratification.

## 5. Conclusions

HFRS is a valid scoring system for the early identification of frail elderly patients at risk of postoperative sepsis. HFRS may be used as an instrument for risk stratification of elderly patients admitted for surgical interventions for vigilant perioperative monitoring and for initiation of timely treatment. As aging frail patients become increasingly common in surgical practice and given that they significantly contribute to hospital mortality and morbidity it is necessary to proactively identify and monitor vulnerable patients with reduced physiological reserves and greater surgical risks. This study offers important results regarding the perioperative surveillance of high-risk frail patients.

## Figures and Tables

**Table 1:** General characteristics of the patients analyzed in the study (*n* = 254,836) and of the patients with incidence of postoperative sepsis population (*n* = 2645).

Variable (Median, IQR) or %	All Patients (*n* = 254,836)	Patients without Postoperative Sepsis (*n* = 252,191, 98.96%)	Patients with Postoperative Sepsis (*n* = 2645, 1.04%)	*p*-Value
Age	83 (79–87)	83 (79–87)	82 (79–86)	<0.001
Sex				<0.001
male	92,245 (36.20%)	90,822 (36.01%)	1423 (53.80%)	
female	162,591 (63.80%)	161,369 (63.99%)	1222 (46.20%)	
ICU	10,213 (4.01%)	8814 (3.49%)	1399 (52.89%)	<0.001
Duration of stay in the ICU (days)	1 (1–4)	1 (1–3)	5 (2–10)	<0.001
Length of stay (days)	7 (4–11)	7 (4–11)	17 (9–30)	<0.001
Elixhauser Comorbidity Index	2 (1–3)	2 (1–3)	3 (2–4)	<0.001
Year				<0.001
2016	84,060 (32.99%)	83,025 (32.92%)	1035 (39.13%)	
2017	86,970 (34.13%)	85,958 (34.08%)	1012 (38.26%)	
2018	83,806 (32.89%)	83,208 (32.99%)	598 (22.61%)	
In-hospital mortality	8217 (3.22%)	6922 (2.74%)	1295 (48.96%)	<0.001
Hospital frailty risk score (HFRS)	1.8 (0–5)	1.8 (0–5)	6.3 (3.5–9.8)	<0.001

ICU: Intensive Care Unit.

**Table 2:** Selected Diagnosis-Related Groups in the patient population (*n* = 254,836).

Types of Surgery	No Sepsis (*n* = 252,191)	Sepsis (*n* = 2645)	Incidence of Postoperative Sepsis (%)
Major stomach, esophageal, and duodenal procedures	4111	339	7.62
Major intestinal procedures	21,337	1277	5.65
Hernia repair	25,706	61	0.24
Laparoscopic cholecystectomy	23,449	422	1.77
Hip replacement	62,455	222	0.35
Knee replacement	41,711	39	0.09
Trauma hip procedures	73,422	285	0.39

**Table 3:** Patient characteristics by Diagnosis-Related Groups (*n* = 254,836).

Diagnosis-Related Groups	Female/Male Ratio	Age, Years Median (25th,75th Percentile)	Mortality	Incidence of Postoperative Sepsis	* ECI, Median (Q1, Q3)	^∞^ HFRS, Median (Q1, Q3)
			%	%		
Major stomach, esophageal, and duodenal procedures	0.85	81 (78–84)	13.48	7.62	3 (2–4)	1.5 (0–3.3)
Major intestinal procedures	0.85	81 (78–85)	9.85	5.65	3 (2–4)	0.9 (0–2.8)
Hernia repair	0.35	81 (78–85)	0.88	0.24	1 (0–2)	0 (0–1.4)
Laparoscopic cholecystectomy	1.18	81 (78–84)	1.26	1.77	2 (1–3)	0 (0–1.5)
Hip replacement	2.32	84 (80–88)	2.95	0.35	2 (1–3)	4.3 (1.4–6.4)
Knee replacement	2.36	79 (77–81)	0.17	0.09	1 (0–2)	0 (0–0.9)
Trauma hip procedures	3.52	86 (82–90)	3.99	0.39	2 (1–3)	4.9 (3.5–7.6)

* ECI—Elixhauser Comorbidity Index; ^∞^ HFRS—Hospital Frailty Risk Score; Q1, Q3: Quartile 1 and 3

**Table 4:** OR of Unadjusted and Adjusted logistic regression models of HFRS as a risk factor for the incidence of postoperative sepsis for different DRGS.

Diagnosis-Related Group	Unadjusted	Adjusted *
	OR (CI 95%)	OR (CI 95%)
Major stomach, esophagus and duodenal procedure	1.36 (1.32, 1.40)	1.09 (1.04, 1.14)
Major procedures: intestine	1.38 (1.36, 1.39)	1.08 (1.06, 1.11)
Hernia procedures	1.68 (1.58, 1.77)	1.18 (1.07, 1.30)
Laparoscopic cholecystectomy	1.54 (1.51, 1.59)	1.13 (1.08, 1.18)
Hip replacement	1.25 (1.23, 1.28)	1.09 (1.06, 1.12)
Knee replacement	1.52 (1.44, 1.61)	1.05 (0.96, 1.15)
Trauma hip procedures	1.27 (1.25, 1.29)	1.07 (1.04, 1.09)

* ECI—Elixhauser Comorbidity Index; HFRS—Hospital Frailty Risk Score; CI 95%: Confidence intervals at 95.

**Table 5:** Adjusted and Unadjusted Multivariable logistic models for the incidence of postoperative sepsis for Elixhauser Comorbidity Index and HFRS and predictive capacity estimated by Area Under the Curve of the different models.

		Unadjusted OR	
Elixhauser Comorbidity Index		1.11 (1.08–1.14) (AUC: 0.896)	
HFRS		1.36 (1.34–1.37) (AUC: 0.933)	
	**Adjusted OR**	**CI95%**	
EIC	1.11	1.09	1.14
HFRS	1.34	1.33	1.36
AUC	0.934		

**Table 6:** Adjusted and Unadjusted Multivariable logistic models for the incidence of postoperative sepsis for Elixhauser and HFRS and predictive capacity estimated by Area Under the Curve of the different models.

HFRS Categorized A							
	**Unadjusted OR**	**CI 95%**			**Adjusted OR**	**CI 95%**	
				EIC	1.06	1.04	1.090
0	Reference			0	Reference		
0.1–4.9	14.38	11.14	18.56	0.1–4.9	6.72	5.07	8.90
5.0-9.9	76.58	59.12	99.18	5.0–9.9	21.43	16.50	27.81
≥10	192.73	146.77	253.07	≥10	89.17	68.8	115.57
AUC	0.937				0.937		
HFRS Categorized B							
	**Unadjusted OR**	**CI 95%**			**Adjusted OR**	**CI 95%**	
				EIC	1.07	1.04	1.09
0	Reference			0	Reference		
0.1–1.7	13.57	10.50	17.53	0.1–1.7	7.03	5.31	9.31
1.8–4.9	69.54	53.55	90.32	1.8–4.9	22.95	17.70	29.74
≥5.0	171.89	130.45	226.50	≥5.0	98.41	76.15	127.16
AUC	0.938				0.937		

EIC: Elixhauser Comorbidity Index; CI 95%: Confidence intervals at 95%; AUC: Area under the Curve.

## Data Availability

Data are available on request.

## Supplementary Materials

The following supporting information can be downloaded at: https://www.mdpi.com/article/10.3390/ijerph20010359/s1, Table S1: List of ICD-10-CM codes, number of points for each to create the Hospital Frailty Risk Score.
