# Supplementary material for: Detection of the Frail Elderly at Risk of Postoperative Sepsis
Source: Int J Environ Res Public Health. 2022 Dec 26;20(1):359. doi: 10.3390/ijerph20010359 (PMC9819229; doi:10.3390/ijerph20010359)
Supplement: Supplementary file 1 [file ijerph-20-00359-s001.zip › ijerph-2101810-supplementary.pdf]

Supplementary Table 1. List of ICD-10-CM codes, number of points for each to create the Hospital Frailty Risk Score

Codes	Definition	Point
**F00**	Dementia in Alzheimer	7·1
**G81**	Hemiplegia	4·4
**G30**	Alzheimer's disease	4·0
**I69**	Sequelae of cerebrovascular disease (secondary codes)	3·7
**R29**	Other symptoms and signs involving the nervous and musculoskeletal systems (R29·6 Tendency to fall)	3·6
**N39**	Other disorders of urinary system (includes urinary tract infection and urinary incontinence)	3·2
**F05**	Delirium, not induced by alcohol and other psychoactive substances	3·2
**W19**	Unspecified fall	3·2
**S00**	Superficial injury of head	3·2
**R31**	Unspecified hematuria	3·0
**B96**	Other bacterial agents as the cause of diseases classified to other chapters (secondary code)	2·9
**R41**	Other symptoms and signs involving cognitive functions and awareness	2·7
**R26**	Abnormalities of gait and mobility	2·6
**I67**	Other cerebrovascular diseases	2·6
**R56**	Convulsions, not elsewhere classified	2·6
**R40**	Somnolence, stupor and coma	2·5

**T83**	Complications of genitourinary prosthetic devices, implants and grafts	2·4
**S06**	Intracranial injury	2·4
**S42**	Fracture of shoulder and upper arm	2·3
**E87**	Other disorders of fluid, electrolyte and acid-base balance	2·3
**M25**	Other joint disorders, not elsewhere classified	2·3
**E86**	Volume depletion	2·3
**R54**	Senility	2·2
**F03**	Unspecified dementia	2·1
**W18**	Other fall on same level	2·1
**Z75**	Problems related to medical facilities and other health care	2·0
**F01**	Vascular dementia	2·0
**S80**	Superficial injury of lower leg	2·0
**L03**	Cellulitis	2·0
**H54**	Blindness and low vision	1·9
**E53**	Deficiency of other B group vitamins	1·9
**G20**	Parkinson's disease	1·8
**R55**	Syncope and collapse	1·8
**S22**	Fracture of rib(s), sternum and thoracic spine	1·8
**K59**	Other functional intestinal disorders	1·8

**N17**	Acute renal failure	1·8
**L89**	Decubitus ulcer	1·7
**Z22**	Carrier of infectious disease	1·7
**B95**	Streptococcus and staphylococcus as the cause of diseases classified to other chapters	1·7
**L97**	Ulcer of lower limb, not elsewhere classified	1.6
**R44**	Other symptoms and signs involving general sensations and perceptions	1·6
**K26**	Duodenal ulcer	1·6
**I95**	Hypotension	1·6
**N19**	Unspecified renal failure	1·6
**A41**	Other septicemia	1·6
**X59**	Exposure unspecified factor	1·5
**Z87**	Personal history of other diseases and conditions	1·5
**J96**	Respiratory failure, not elsewhere classified	1·5
**M19**	Other arthrosis	1·5
**G40**	Epilepsy	1·5
**M81**	Osteoporosis without pathological fracture	1·4
**S72**	Fracture of femur	1·4
**S32**	Fracture of lumbar spine and pelvis	1·4
**E16**	Other disorders of pancreatic internal secretion	1·4

**R94**	Abnormal results of function studies	1·4
**N18**	Chronic renal failure	1·4
**R33**	Retention of urine	1·3
**R69**	Unknown and unspecified causes of morbidity	1·3
**N28**	Other disorders of kidney and ureter, not elsewhere classified	1·3
**G31**	Other degenerative diseases of nervous system, not elsewhere classified	1·2
**R32**	Unspecified urinary incontinence	1·2
**S09**	Other and unspecified injuries of head	1·2
**R45**	Symptoms and signs involving emotional state	1·2
**G45**	Transient cerebral ischemic attacks and related syndromes	1·2
**Z74**	Problems related to care-provider dependency	1·1
**M79**	Other soft tissue disorders, not elsewhere classified	1·1
**W06**	Fall involving bed	1.1
**S01**	Open wound of head	1.1
**A09**	Diarrhea and gastroenteritis of presumed infectious origin	1.1
**J18**	Pneumonia, organism unspecified	1·1
**A04**	Other bacterial intestinal infections	1·1
**R02**	Gangrene not classified anywhere	1·0
**J69**	Pneumonitis due to solids and liquids	1·0

**R47**	Speech disturbances, not elsewhere classified	1·0
**E55**	Vitamin D deficiency	1·0
**Z93**	Artificial opening status	1·0
**R63**	Symptoms and signs concerning food and fluid intake	0·9
**H91**	Other hearing loss	0·9
**W10**	Fall on and from stairs and steps	0·9
**W01**	Fall on same level from slipping, tripping and stumbling	0·9
**E05**	Thyrotoxicosis [hyperthyroidism]	0·9
**M41**	Scoliosis	0·9
**R13**	Dysphagia	0·8
**Z99**	Dependence on enabling machines and devices	0·8
**M80**	Osteoporosis with pathological fracture	0·8
**K92**	Other diseases of digestive system	0·8
**I63**	Cerebral Infarction	0·8
**N20**	Calculus of kidney and ureter	0·7
**F10**	Mental and behavioral disorders due to use of alcohol	0·7
**Y84**	Other medical procedures as the cause of abnormal reaction of the patient	0·7
**R00**	Abnormalities of heart beat	0·7
**J22**	Unspecified acute lower respiratory infection	0·7
**Z73**	Problems related to life-management difficulty	0·6

**Z91**	Personal history of risk-factors, not elsewhere classified	0·5
**S51**	Open wound of forearm	0·5
**F32**	Depressive episode	0·5
**M48**	Spinal stenosis	0·5
**E83**	Disorders of mineral metabolism	0·4
**M15**	Polyarthrosis	0·4
**D64**	Other anemias	0·4
**L08**	Other local infections of skin and subcutaneous tissue	0·4
**R11**	Nausea and vomiting	0·3
**K52**	Other noninfective gastroenteritis and colitis	0·3
**R50**	Fever of unknown origin	0·1
**Z60**	Problems related to social environment	0
**R79**	Other abnormal findings of blood chemistry	0
**U80**	Agent resistant to penicillin and related antibiotics	0
